# BK Polyomavirus-specific T cell immune responses in kidney transplant recipients diagnosed with BK Polyomavirus-associated nephropathy

**DOI:** 10.1186/s12879-019-4615-x

**Published:** 2019-11-19

**Authors:** Jackrapong Bruminhent, Supranart Srisala, Chompunut Klinmalai, Subencha Pinsai, Siriorn P. Watcharananan, Surasak Kantachuvesiri, Suradej Hongeng, Nopporn Apiwattanakul

**Affiliations:** 1 Division of Infectious Diseases, Department of Medicine, Faculty of Medicine Ramathibodi Hospital, Mahidol University, Bangkok, Thailand; 2 Excellence Center of Organ Transplantation, Faculty of Medicine Ramathibodi Hospital, Mahidol University, Bangkok, Thailand; 3 Research Center, Faculty of Medicine Ramathibodi Hospital, Mahidol University, Bangkok, Thailand; 4 Division of Infectious Diseases, Department of Pediatrics, Faculty of Medicine Ramathibodi Hospital, Mahidol University, Bangkok, Thailand; 5 Division of Nephrology, Department of Medicine, Faculty of Medicine Ramathibodi Hospital, Mahidol University, Bangkok, Thailand; 6 Division of Hematology and Oncology, Department of Pediatrics, Faculty of Medicine Ramathibodi Hospital, Mahidol University, Bangkok, Thailand

**Keywords:** BKPyV, BKVAN, BKPyVAN, T cell immunity, Immune monitoring, Intracellular cytokine assay

## Abstract

**Background:**

Adjustment of immunosuppression is the main therapy for BK polyomavirus (BKPyV)-associated nephropathy (BKPyVAN) after kidney transplantation (KT). Studies of BKPyV-specific T cell immune response are scarce. Here, we investigated BKPyV-specific T cell immunity in KT recipients diagnosed with BKPyVAN.

**Methods:**

All adult KT recipients with BKPyVAN diagnosed at our institution from January 2017 to April 2018 were included. Laboratory-developed intracellular cytokine assays measuring the percentage of IFN-γ-producing CD4^+^ and CD8^+^ T cells, after stimulation with large-T antigen (LT) and viral capsid protein 1 (VP1), were performed both at the time of diagnosis and after adjustment of immunosuppression.

**Results:**

We included 12 KT recipients diagnosed with BKPyVAN (7 proven, 4 presumptive, and 1 possible). Those with presumptive BKPyVAN had a median plasma BKPyV DNA load of 5.9 log10 copies/ml (interquartile range [IQR]: 4.9–6.1). Adjusted dosing of mycophenolic acid and tacrolimus with (86%) or without (14%) adjunctive therapies were implemented after diagnosis. There was a significantly higher median percentage of IFN-γ-producing CD4^+^ T cells to LT at a median of 3 (IQR: 1–4) months after adjustment of immunosuppression compared with at the time of diagnosis (0.004 vs. 0.015; *p* = 0.047). However, the difference between the median percentage of IFN-γ-producing CD4^+^ T cells to VP1 and CD8^+^ T cells to LT and VP1 did not reach statistical significance. Four (33%) patients achieved plasma BKPyV DNA clearance, and the remaining eight (67%) patients had persistent BKPyV DNAemia. Although eight (67%) patients developed allograft dysfunction, none required hemodialysis.

**Conclusions:**

We observed a marginal trend of BKPyV-specific CD4^+^ T cell recovery after adjustment of immunosuppression in KT recipients diagnosed with BKPyVAN. A further study would be benefited to confirm and better assess BKPyV-specific immune response after KT.

## Background

BK polyomavirus (BKPyV)-associated nephropathy (BKPyVAN) is one of the major causes of allograft dysfunction after kidney transplantation (KT). The incidence of BKPyVAN is reported to be 8%, ranging from 1 to 10% after KT [1, 2]. A recent retrospective study at our institution, a resource-limited setting, revealed a relatively high BKPyVAN rate compared with those reported in the literature. BKPyVAN was found to be associated with allograft failure, independent of other confounding factors [3]. Because BKPyV DNAemia is a surrogate marker of an over-immunosuppressed state in KT recipients, and no proven anti-BKPyV agents are currently available, adjustment of immunosuppression is considered the main therapy for BKPyVAN. Cellular adaptive immunity, including CD4^+^ and CD8^+^ T cells, plays an essential role in the control of BKPyV [4]. A lack of BKPyV-specific T cell immunity has been shown to be a risk factor for BKPyVAN [2, 5]. BKPyV-specific cellular immune recovery after an adjustment of immunosuppression has been proposed as a strategy to achieve BKPyV clearance. Furthermore, immune-based therapies to control BKPyV may provide an opportunity for the successful treatment of BKPyVAN [6]. Although studies have investigated BKPyV-specific T cell immunity in hematopoietic stem cell transplant (HSCT) recipients, work focused on this factor in solid organ transplant (SOT) recipients have been limited. A low BKPyV-specific T cell immunity prior to transplant has been reported by several studies as a predictor for BKPyV DNAuria and/or DNAemia after KT [7–9]. However, the studies which focused on BKPyV-specific immunity after adjustment of immunosuppression are scarce. Here, we performed a study to investigate BKPyV-specific T cell immunity in KT recipients with BKPyVAN, both at the time of diagnosis and after adjustment of immunosuppression, to assess immune response in this specific infection. The objective of this studies was to investigate a role of BKPyV-specific T cell immunity by measuring BKPyV-specific CD4+ and CD8+ T responses in KT recipients diagnosed with BKPyVAN at the time of diagnosis and after adjustment of immunosuppression.

## Methods

### Subjects

We included all adult KT recipients diagnosed with BKPyVAN from January 2017 to April 2018 at a single transplant center in Bangkok, Thailand. All patients with increased serum creatinine were investigated for BKPyVAN. Demographic, virological, and immunological data were collected, and the outcomes of plasma BKPyV DNA clearance and kidney allograft function were assessed. BKPyVAN was defined in accordance with current guidelines [1] as being either “proven” (presence of viral cytopathic changes on histopathology and positive staining of polyomavirus simian virus (SV) 40 large-T antigen (LT) via immunohistochemistry), “presumptive” (presence of plasma BKPyV DNA load of 4.0 log10 copies/ml or higher), or “possible” (presence of urine BKPyV DNA load of 7.0 log10 copies/ml or higher). Plasma and urine BKPyV DNA loads were measured by quantitative real-time polymerase chain reaction (PCR) assays (Vela Diagnostics; Fairfield, NJ, USA). The BKPyV DNA load was reported in copies/ml with a limit of quantification of 187–10^8^ copies/ml or 2.2–8.0 log10 copies/ml. The plasma BKPyV DNA load was measured approximately every 2 weeks after adjustment of immunosuppression. BKPyV DNA clearance was defined as the achievement of two consecutive undetectable BKPyV DNA loads in urine or plasma. Allograft dysfunction was defined as unrecovered estimated glomerular filtration rate reduction after infection.

According to current guidelines [1], adjustment of immunosuppression includes the reduction or discontinuation of mycophenolic acid, e.g., keeping mycophenolate mofetil levels of < 1.0 g/day and maintaining tacrolimus and cyclosporine C_0_ concentration of < 6 and 150 ng/ml, respectively. Tacrolimus was switched to either cyclosporine or sirolimus. If DNA clearance was not achieved, the transplant nephrologist or infectious diseases specialist made a decision regarding the need for adjunctive therapies on an individual basis. The adjunctive therapies included intravenous immunoglobulin (IVIG) at a dose of 2 g/kg divided over 4–5 days, intravenous cidofovir at a dose of 0.5–1 mg/kg weekly, or oral leflunomide at a dose of 100 mg/day for the first 5 days followed by 40 mg/day. The latter two therapies were continued until DNA clearance was achieved or a persistent level of BKPyV DNAemia (in cases without clearance) was observed.

### Intracellular IFN-γ measurement of BKPyV-specific T cell immunity

Laboratory-developed intracellular cytokine assays (ICAs) measuring the percentage of IFN-γ-producing CD4^+^ and CD8^+^ T cells, using LT and VP1, were performed both at the time of diagnosis and after adjustment of immunosuppression. The BKPyV-specific T cells were the cells which were able to produce IFN-γ upon stimulation with LT or VP1. Both LT and VP1 antigens were accessed from JPT Peptide Technologies (Berlin, Germany). Peripheral blood mononuclear cells (PBMCs) were separated by density gradient techniques. Isolated PBMCs, prepared in RPMI1640 medium, were activated by specific peptides (LT or VP1: 1 μg/ml.). Cells were then further incubated at 37 °C with 5% CO_2_ for 18–24 h. A block of cytokine secretion and intracellular accumulation were achieved by treatment with brefeldin A (Biolegend, Inc., San Diego, CA, USA). At 3 h after the brefeldin A stimulation, the cells were fixed with 2% formaldehyde (Sigma-Aldrich, Inc., St. Louise, MO, USA) and permeabilized with 0.1% saponin (Sigma-Aldrich, Inc.) before being stained with fluorescent antibodies directed against the cytokine and finally analyzed by flow cytometry. The BKPyV-specific T cell subset was identified by the direct immunofluorescence of the following monoclonal antibodies (eBioscience Inc. San Diego, CA, USA): anti-CD3 (labeled with FITC), anti-CD4 (labeled with APC), anti-CD8 (labeled with APC efluor780), and anti-IFN-γ (labeled with PECy7). After being left to incubate with a cocktail of these antibodies for 30 min at 4 °C in the dark, the cells were analyzed using FACSVerse (BD Pharmingen. Franklin Lakes, NJ, USA) and Flowjo software (Flowjo, LLC; Ashland, OR, USA). The results are expressed as the percentages of cells within the total population.

### Ethics

The study was approved by the Institutional Review Board of the Faculty of Medicine Ramathibodi Hospital, Mahidol University, Bangkok, Thailand with the provisions of the Good Clinical Practice Guidelines and the Declaration of Helsinki. All patients provided written informed consent prior to participation.

### Statistical analysis

The clinical characteristics of the patients were analyzed by descriptive analyses. Categorical and continuous data were described as absolute and relative frequencies and as medians with interquartile ranges (IQRs), respectively. The percentages of IFN-γ-producing CD4^+^ and CD8^+^ T cells at the time of diagnosis were compared with those after adjustment of immunosuppression by non-parametric Wilcoxon signed-rank tests, and *p*-values of < 0.05 produced by two-tailed tests were considered statistically significant.

## Results

### Demographic data

We included 12 KT recipients with a median age of 42 (IQR: 35–50) years, and 67% of them were male. Seven (58%) patients underwent deceased-donor KT. Furthermore, eight (67%) received induction therapy (anti-thymocyte globulin, *n* = 1; interleukin-2 receptor antagonist, *n* = 7), followed by maintenance therapy (tacrolimus, 100%; mycophenolate mofetil, 83%; mycophenolate sodium, 17%; and prednisolone, 100%). Antimicrobial chemoprophylaxis included trimethoprim/sulfamethoxazole for *Pneumocystis jirovecii*, acyclovir for herpes simplex virus, and isoniazid for latent tuberculous infection. All patients had both donor and recipient cytomegalovirus (CMV) seropositivity. They underwent preemptive monitoring instead of anti-CMV prophylaxis.

### Diagnosis and management of BKPyVAN

Of the 12 KT recipients with BKPyVAN in our study, there were seven proven cases, four presumptive cases, and one possible case. The median time to BKPyVAN diagnosis was 9 (IQR: 4.5–12) months; 8 (67%) patients developed BKPyVAN within 12 months post-KT. Those with proven BKPyVAN had detectable cytopathic changes and positive SV 40 staining. Those with presumptive BKPyVAN had a median plasma BKPyV DNA load of 5.9 (IQR: 4.9–6.1) log10 copies/ml. The one patient with possible BKPyVAN had a urine BKPyV DNA load of > 8.0 log10 copies/ml. Details of the adjusted immunosuppression implemented in each patient are shown in Table 1**.** All patients underwent discontinuation (*n* = 7) or reduction (*n* = 3) of mycophenolate mofetil with a median dose reduction of 1.1 (IQR: 0.8–1.5) g/day, and the later were maintained at a dosing level of < 1.0 g/day. Mycophenolate sodium was discontinued for the remaining patients (*n* = 2). All patients were maintained at tacrolimus C_0_ concentration of 3–5 ng/ml; five of these patients were later switched to cyclosporine (*n* = 4) and sirolimus (*n* = 1). Prednisolone was maintained at 5 mg/day in ten of the patients. Adjunctive therapies included cidofovir (*n* = 6), IVIG (n = 4), and leflunomide (*n* = 9).

**Table 1:** Management and outcome of 12 kidney transplant recipients with BK polyomavirus-associated nephropathy

No.	Diagnosis of BKPyVAN	Adjustment of immunosuppression			Adjunctive therapies			BKPyV clearance	Allograft dysfunction
		Mycophenolic acid	Tacrolimus C_0_ level of 3–5 ng/mL	Switched tacrolimus to cyclosporine	Cidofovir	IVIG	Leflunomide		
1	Presumptive	Discontinuation	Yes	No	Yes	No	Yes	Yes	Yes
2	Proven	Discontinuation	Yes	Yes	No	Yes	Yes	No	Yes
3	Presumptive	Reduction	Yes	Yes	Yes	Yes	Yes	Yes	No
4	Presumptive	Discontinuation	Yes	No	Yes	No	Yes	No	Yes
5	Proven	Discontinuation	Yes	No	Yes	No	No	No	Yes
6	Possible	Reduction	Yes	No	No	No	No	Yes	No
7	Proven	Discontinuation	Yes	Yes	Yes	Yes	Yes	No	Yes
8	Presumptive	Reduction	Yes	No	No	No	Yes	Yes	Yes
9	Proven	Discontinuation	Yes	Yes	No	Yes	Yes	No	Yes
10	Proven	Discontinuation	Yes	No	No	No	Yes	No	Yes
11	Proven	Discontinuation	Yes	Yes (sirolimus)	Yes	No	Yes	No	No
12	Proven	Discontinuation	Yes	No	No	No	No	No	No

*BKPyV* BK polyomavirus, *BKPyVAN* BK polyomavirus-associated nephropathy, *IVIG* intravenous immunoglobulin

Four (33%) patients achieved plasma BKPyV DNA clearance, whereas the other eight (67%) patients still had persistent BKPyV DNAemia. One (25%) of the patients who achieved BKPyV DNA clearance later developed a recurrent low-level BKPyV DNAemia (< 1000 copies/ml) without allograft dysfunction after the resumption of immunosuppression. Additionally, one (8%) patient developed biopsy-proven acute cellular rejection after adjustment of immunosuppression. Eight (67%) patients developed allograft dysfunction at the end of management. No patient required hemodialysis or died during the study period.

### BKPyV-specific T cell immune responses

**Fig. 1:**
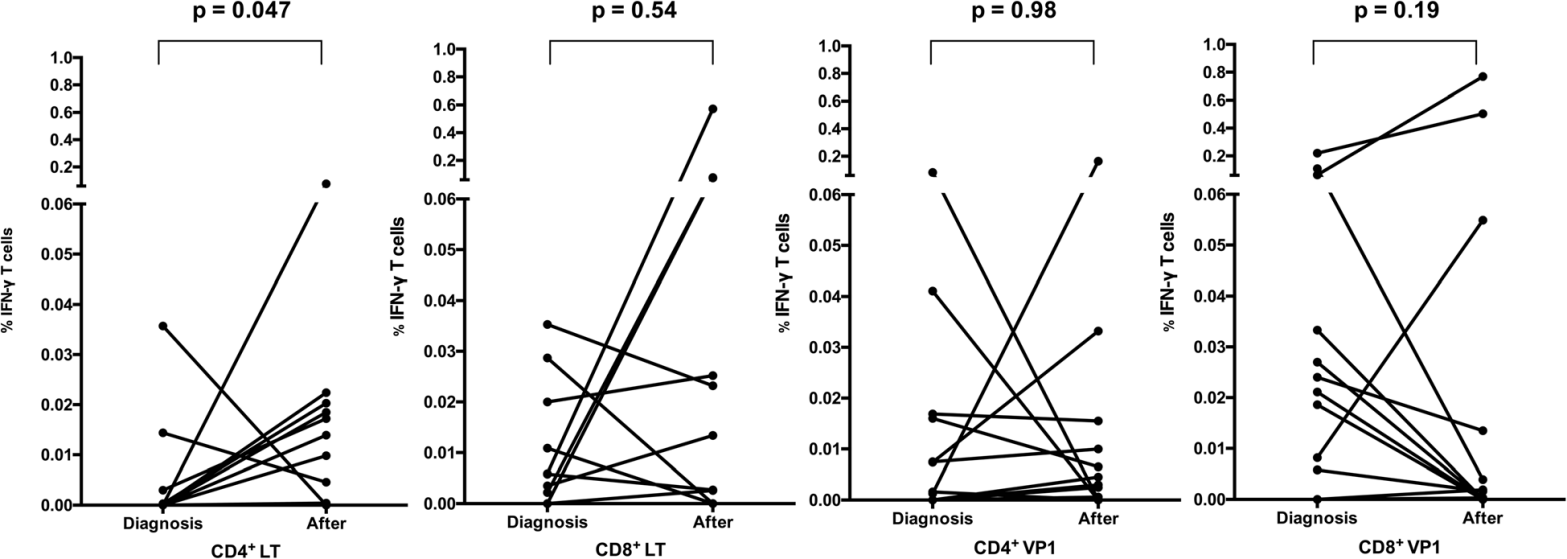
Percentage of IFN-γ-producing BKPyV-specific T cells in 12 KT recipients with BKPyVAN. The median percentages of IFN-γ-producing BKPyV-specific CD4^+^ and CD8^+^ cells stimulated by LT and VP1 antigens at diagnosis and after adjustment of immunosuppression were compared

**Fig. 2:**
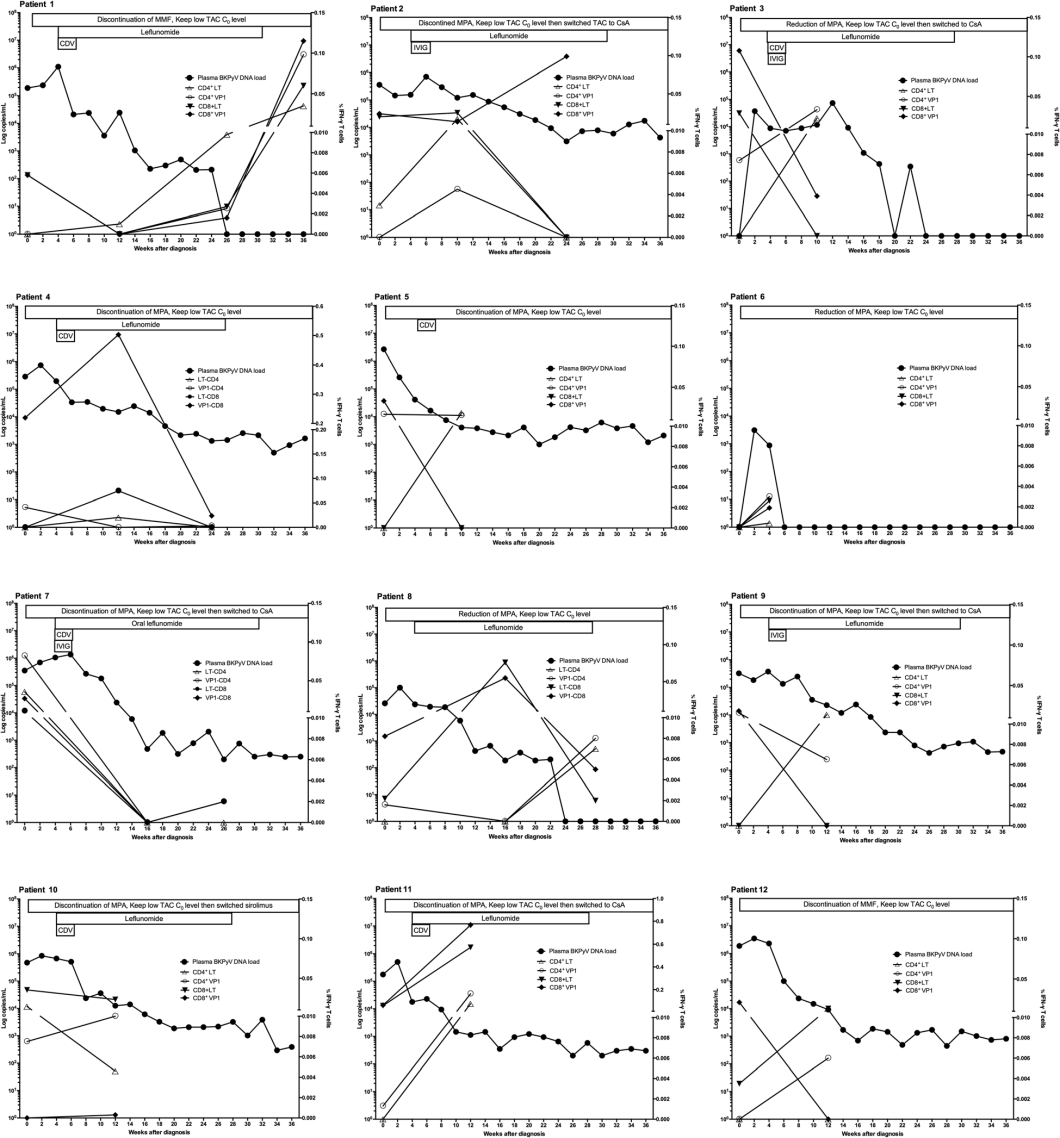
The correlation of plasma BKPyV DNA load (log10 copies/ml), as well as LT, and VP1-specific CD4^+^ and CD8^+^ T cell responses measured by intracellular cytokine assay of each patient; patient number 1, 2, 4, 7 and 8 who underwent dynamic measurements at additional time points were also shown. Those are expressed as the percentage of IFN-γ cells related to the percentage of cells in the total population. The horizontal bar indicates the start of intervention as indicated; MPA, mycophenolic acid; TAC, tacrolimus; CsA, cyclosporine A; IVIG, intravenous immunoglobulin

**Fig. 3:**
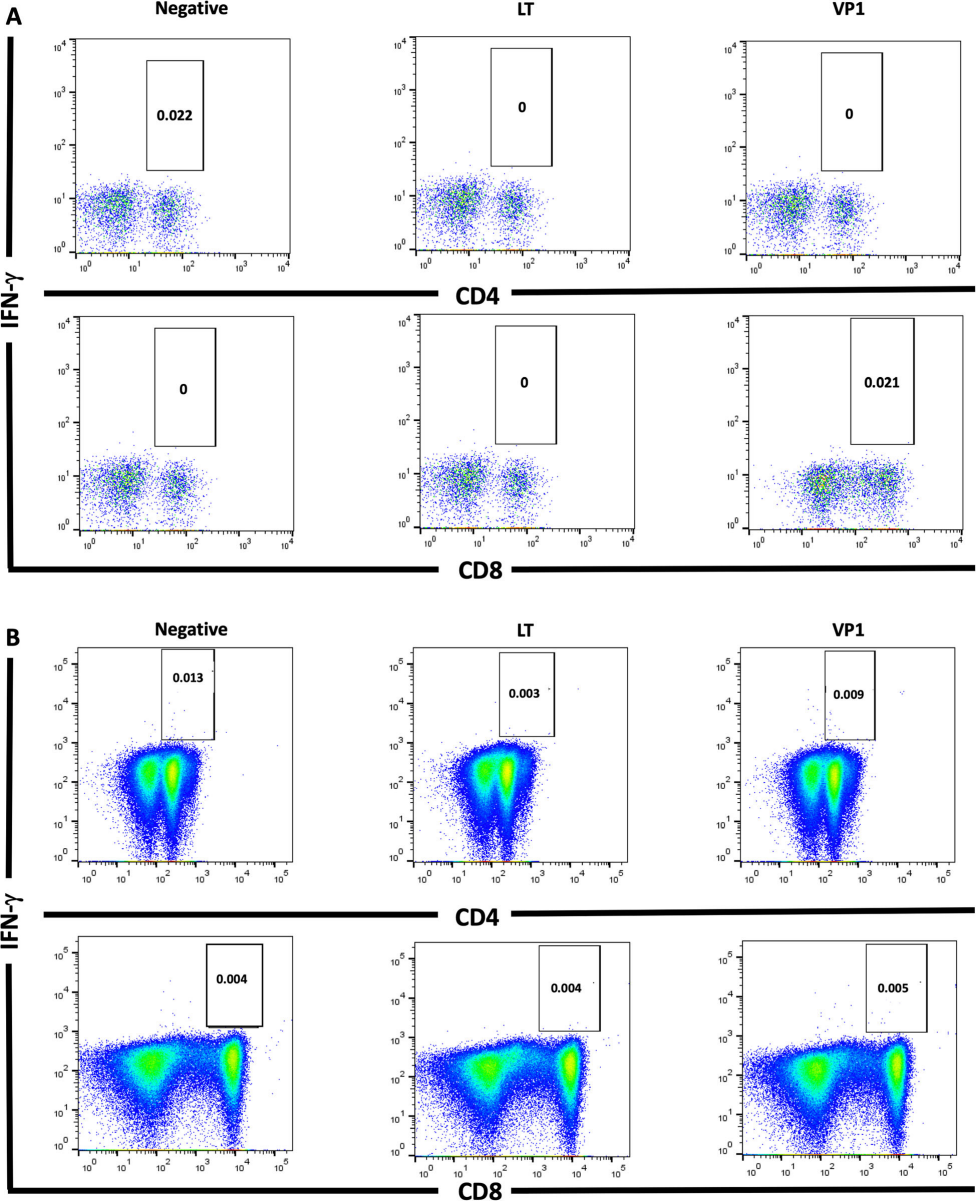
BKPyV-specific CD4^+^ (upper panel) and CD8^+^ (lower panel) T cells with IFN-γ production in KT recipient; patient number 8. Peripheral blood mononuclear cells were tested by intracellular cytokine staining for production of IFN-γ after stimulation with LT and VP1 antigens at diagnosis (**a**) and after adjustment of immunosuppression (**b**). These IFN-γ-producing cells were determined on the basis of their forward and side scatter plots

All patients underwent measurement of IFN-γ-producing CD4^+^ and CD8^+^ T cells by ICA after stimulation by LT and VP1, both at the time of BKPyVAN diagnosis and at a median of 3 (IQR: 1–4) months after adjustment of immunosuppression (Fig. 1). The median percentage of IFN-γ-producing CD4^+^ T cells to LT after adjustment of immunosuppression was significantly higher than that at the time of diagnosis (0.004 vs. 0.015; *p* = 0.047). Although the median percentages of IFN-γ-producing CD8^+^ T cells to LT was slightly higher after adjusting the immunosuppression than at the time of diagnosis, this difference did not reach statistical significance. Furthermore, there was no difference of IFN-γ-producing CD4^+^ & CD8^+^ T cells to VP1 between at the time of diagnosis and after adjustment of immunosuppression. We also presented the BKPyV-specific T cell response along with plasma BKPyV DNA DNA monitoring of each patient in Fig. 2; patient number 1, 2, 4, 7 and 8 who underwent dynamic measurements at additional time points were also shown. Intracellular flow cytometry was used to determine IFN-γ production in BKPyV-specific CD4^+^ and CD8^+^ T cells in one representative patient; patient number 8 are shown in Fig. 3**.**

## Discussion

Here, we report a study measuring BKPyV-specific T cell immunity in KT recipients at the time of BKPyVAN diagnosis as well as after adjustment of immunosuppression. Our results show an increased percentage of IFN-γ-producing CD4^+^ T cells after stimulation with LT as measured by ICA, suggesting a trend of BKPyV-specific cellular immune recovery.

A role for virus-specific immune monitoring of certain viral infections in SOT recipients has been recently proposed [10]. A lack of virus-specific T cell quantity or functionality has been reported as a risk factor of viral infection after KT. To date, most investigations have applied CMV-specific immunity assays. Patients with decreased or absent CMV-specific T cell immunity, as measured by an ELISpot, were shown to be at risk of CMV infection after KT [11]. Notably, CD8^+^ T cell responses determined via QuantiFERON-CMV assays have been utilized in clinical practice to predict which patients are at risk of recurrent infection after treatment [12]. CMV-specific immunity assays have been recommended for use in the management and prevention of CMV infection in SOT recipients in a recently updated set of international guidelines [13].

The potential role of BKPyV-specific T cell immunity has been more investigated in HSCT recipients. BKPyV-specific CD4^+^ and CD8^+^ T cell recovery were associated with successful BKPyV clearance in pediatric HSCT recipients with a diagnosis of BKPyV-associated hemorrhagic cystitis [14]. Few studies focusing on this factor in KT recipients have been reported. Dewolfe et al. found that low levels of CD4^+^ T cells, high levels of CD8^+^ T cells, and increased levels of effector CD8^+^ T cells prior to KT were all associated with BKPyV DNAemia after KT [9]. Schachtner et al. also reported that KT recipients who failed to develop BKPyV-specific T cell immunity prior to KT tended to develop BKPyV DNAemia after KT [8]. In contrast with the aforementioned studies, both of which identified pre-KT immunological predictors, our study instead focused on post-KT predictors after adjustment of immunosuppression. We found that the CD4^+^ T cell response after stimulation with LT was significantly recovered after adjustment of immunosuppression. However, the CD4^+^ T cell response to VP1 and CD8^+^ T cell responses to LT and VP1 did not reach statistical significance.

Our data support the theory that adaptive cellular immunity may have a potential role in BKPyV control. In healthy patients, T cell-mediated immunity to BKPyV is associated with the multifunctional properties of CD4^+^ T cells, as both T-helper and T-cytotoxic cells, for BKPyV clearance [15]. Weist et al. reported an important role for BKPyV-specific CD4^+^ T cells as a dominant population that reconstitutes itself to control BKPyV in KT recipients [16]. Binggeli et al. reported significantly higher BKPyV-specific IFN-γ responses in KT recipients who achieved decreased or cleared BKPyV DNA loads compared with those who had increased or persistent BKPyV DNA loads. However, in contrast to our results, they found VP1-specific IFN-γ responses were higher and more likely to involve CD4^+^ T cells compared with LT-specific IFN-γ responses, while CD8^+^ T cells were more frequently directed against LT than against VP1 [17]. A significant increase in CD4^+^ T cell responses could be explained from increasing B cells which later stimulate CD4^+^ T cells. Schmidt et al. reported higher BKPyV IgG levels were associated with BKPyV-specific CD4^+^ T cell responses in KT recipients who achieved plasma BKPyV DNA clearance [18]. Furthermore, Leboeuf et al. reported shorter peptide (9mP)-responses may be better reconstituting CD8 T cell responses compared to a longer peptide (15mP) in KT recipients who achieved BKPyV clearance [7]. In our study, both LT and VP1 antigens used in our study were a 15mer peptide which could limit CD8 T cell responses in our study.

The optimal dosing of treatment immunosuppression could be key for the BKPyV-specific immune recovery in our study. Trough levels of tacrolimus were kept between 3 and 5 ng/ml, and mycophenolate mofetil levels were maintained at < 1.0 g/day in our patients. Egli et al. reported a dose-dependent decrease in the IFN-γ production of CD4^+^ and CD8^+^ (slightly more) T cells in response to tacrolimus, but not to mycophenolate mofetil, in vitro [19]. Skulratanasak et al. recently reported KT recipients who received mycophenolic acid of greater than 1.0 g/day was independently associated with BKPyV infection [20]. In vivo, KT recipients with tacrolimus levels of < 6 ng/ml seemed to have significantly higher levels of BKPyV-specific T cell immunity as measured by ELISpot assay compared with those who were kept at levels of > 6 ng/ml [19]. Furthermore, few of our patients were switched to sirolimus or leflunomide. The direct effects of sirolimus and leflunomide on BKPyV inhibition have been debated. However, neither has been shown to inhibit BKPyV-specific IFN-γ production in vitro [19]. Interestingly, the neutralizing and immunomodulatory effects of IVIG were shown to contribute to the resolution of active disease at equivalent levels to the anti-DNA viral activity of cidofovir [1].

We are aware of several limitations in our study. First, this study was based on a small group of patients. A larger study would be helpful to confirm our results. Second, as a BKPyVAN screening protocol was not universally utilized at our institution, our patients were diagnosed relatively late and at different stages of infection as well as variability time of immune measurement after adjustment of immunosuppression. However, BKPyV was reported to be cleared with a viral half-life of 6 h up to 17 days after changing immunosuppressive regimens in KT which could allow us to compare of the immunity during 2–3 weeks period [21]. Last, the direct effects of adjustment of immunosuppression and adjunctive therapies to BKPyV-specific immune recovery could not be investigated. However, maintenance therapy (all patients on tacrolimus) and an adjustment protocol (at least prior to adjunctive therapies) were implemented for all patients to limit this variability of confounding factors among patients. We believe these data likely represent a real-world practice where multi-interventions are commonly implemented as recommended by current guidelines [1]. For the aforementioned reasons, evaluation of immune recovery after adjustment of immunosuppression as a whole group could be limited. Instead, the immune response would be more practical when monitoring on an individual basis. The adjustment of an immunosuppression protocol should be harmonized to evaluate the true effect of BKPyV-specific T cell recovery at each center. Although viral-specific T cell immunity can be measured by various methods, including ICA, ELISpot assay, or ELISA, the use of ICA in our study allowed a deeper exploration of intracellular interferon production in both CD4^+^ and CD8^+^ T cells compared with the alternative methods. However, as a commercial assay for measuring BKPyV-specific T cell immunity is not yet available, this immune-guided strategy will likely be limited to large academic centers for now.

## Conclusions

Our study showed a marginal trend of BKPyV-specific CD4^+^ T cell immune recovery after adjustment of immunosuppression after KT. A larger study would be benefited to confirm and better assess this viral-specific immune response in KT recipients.

## Data Availability

The datasets analyzed during the current study available from the corresponding author on reasonable request.

## Abbreviations

BKPyV: BK polyomavirus
BKPyVAN: BK polyomavirus-associated nephropathy
CMV: Cytomegalovirus
HSCT: Hematopoietic stem cell transplant
ICAs: Intracellular cytokine assays
IQR: Interquartile range
IVIG: Intravenous immunoglobulin
KT: Kidney transplantation
LT: Large-T antigen
PBMCs: Peripheral blood mononuclear cells
PCR: Polymerase chain reaction
SOT: Solid organ transplant
SV: Simian virus
VP1: Viral capsid protein 1
